# Associations of BCW-informed early rehabilitation nursing with patient compliance and related outcomes in non-dialysis chronic kidney disease patients

**DOI:** 10.3389/fpubh.2026.1861842

**Published:** 2026-10-22

**Authors:** Yingxing Zhou, Xiaodong Cao, Xiaomin Xu, Linjie Xu, Xiao Li, Qin Xu

**Affiliations:** 1 Department of Nursing, Wuxi People's Hospital Affiliated to Nanjing Medical University, Wuxi, Jiangsu Province, China; 2 Department of Nursing, School of Nursing, Nanjing Medical University, Wuxi, Jiangsu Province, China

**Keywords:** BCW theory, dietary compliance, early rehabilitation nursing, non-dialysis chronic kidney disease, patient self-management

## Abstract

**Objective:**

To examine the association between a BCW-informed intensive nursing support package and patient compliance and related outcomes in non-dialysis patients with chronic kidney disease (CKD).

**Methods:**

This retrospective, non-randomized observational study analyzed the clinical and nursing records of 57 non-dialysis CKD patients admitted to our hospital from June 2022 to June 2023. All patients met the predefined inclusion and exclusion criteria. Patients were retrospectively classified according to the nursing intervention documented in their clinical records: 27 patients who received routine nursing were included in the control group, and 30 patients who received a BCW-informed intensive nursing support package were included in the observation group. Outcomes included nutrition-related indicators [albumin (ALB), prealbumin (PA), transferrin (TF), and body mass index (BMI)], renal function-related indicators [blood urea nitrogen (BUN), serum creatinine (Scr), and cystatin C (CysC)], self-reported dietary adherence assessed using an adapted Renal Adherence Behavior Questionnaire (RABQ), quality of life, and reported nursing satisfaction.

**Results:**

Before the intervention, there were no significant differences between the two groups in nutrition-related indicators, renal function-related indicators, RABQ scores, or quality of life (all *p* > 0.05). After the intervention, the observation group showed significantly higher ALB, PA, and BMI levels than the control group (*p* < 0.05), whereas the between-group difference in TF was not statistically significant (*p* = 0.169). For renal function-related indicators, BUN was significantly higher and CysC was significantly lower in the observation group than in the control group (*p* < 0.05), whereas Scr was numerically higher but the between-group difference did not reach statistical significance (*p* = 0.054), indicating inconsistent changes among renal function-related markers. After the intervention, the observation group also had higher adapted RABQ scores, indicating higher self-reported dietary adherence on this instrument, and higher quality-of-life scores than the control group (*p* < 0.05). The reported nursing satisfaction rate was 77.78% in the control group and 100.00% in the observation group.

**Conclusion:**

In this retrospective, non-randomized study, the BCW-informed intensive nursing support package was associated with more favorable nutrition-related indicators, higher self-reported dietary adherence scores on the adapted RABQ, better quality-of-life scores, and higher reported nursing satisfaction. Renal function-related indicators were inconsistent, and because the observation group received greater nursing contact, the specific effect of BCW/COM-B cannot be distinguished from nonspecific attention or intervention-dose effects.

## 1 Introduction

Chronic Kidney Disease (CKD) represents a global public health issue, with its prevalence and mortality continuously rising over the past few decades. CKD is classified into five stages based on glomerular filtration rate, and by the time it progresses to stage 5, patients have entered end-stage renal disease and require timely renal replacement therapy (1). Non-dialysis CKD patients refer to individuals diagnosed with CKD who have not yet reached the stage requiring dialysis treatment. These patients typically experience varying degrees of decline in kidney function but have not reached the stage necessitating dialysis. They might face a spectrum of kidney-related issues such as hypertension, anemia, abnormalities in bone metabolism, and electrolyte imbalances (2). Physicians usually take measures to slow down the deterioration of kidney function and manage these patients through medication, dietary management, and lifestyle adjustments (3). In the management of non-dialysis CKD patients, dietary and nutritional management, including nutritional assessment, medical nutrition therapy, and individualized guidance on dietary protein, energy intake, sodium, and other nutrients, is an important component of care. However, due to the complexity of dietary needs and variations in patients’ understanding of nutrition, many patients have suboptimal dietary management, which may affect disease management and complication prevention (4). Therefore, the development of a more scientific and systematic nutritional rehabilitation nursing intervention model holds significant clinical importance.

The Behavior Change Wheel (BCW) was originally proposed by Michie, van Stralen and West as a framework for characterizing and designing behavior change interventions (5). At the center of the BCW is the COM-B model, in which behavior is understood as the result of interactions among capability, opportunity, and motivation. Capability includes physical and psychological capacities, opportunity includes physical and social opportunities, and motivation includes reflective and automatic motivational processes. The BCW also includes nine intervention functions, namely education, persuasion, incentivization, coercion, training, restriction, environmental restructuring, modeling, and enablement. Previous studies have applied the BCW to guide theory-informed health behavior interventions in clinical and public health settings (6). In the present study, the BCW and COM-B model were used as a guiding framework to organize routine nursing education, dietary guidance, medication reminders, peer support, and follow-up support for non-dialysis CKD patients. However, the intervention should be regarded as BCW-informed rather than a full implementation of the complete BCW intervention development process. Specifically, the intervention was not developed through a formal, prospectively conducted COM-B diagnostic study or a structured APEASE scoring process; instead, COM-B and APEASE principles were used pragmatically to refine and classify nursing activities that were feasible in routine clinical practice. Although the BCW has been increasingly used in health behavior promotion, its application in the early rehabilitation nursing of non-dialysis CKD patients remains relatively limited. Therefore, this study explored a BCW-informed early rehabilitation nursing model for non-dialysis CKD patients.

## 2 Objects and methods

### 2.1 Study subjects

This study was a retrospective, non-randomized, observational comparative study. After ethics approval, the clinical and nursing records of non-dialysis CKD patients admitted to our hospital from June 2022 to June 2023 were reviewed. The nursing interventions were not prospectively assigned by the research team for study purposes. Instead, patients were retrospectively classified according to the nursing care model documented in their medical and nursing records.

The inclusion criteria were as follows: ① age ≥18 years; ② diagnosis of CKD based on clinically relevant examinations; ③ meeting the diagnostic criteria for CKD stages 1–5 (7) and having never received dialysis treatment; and ④ complete and reliable clinical data available for analysis. The exclusion criteria were as follows: ① history of kidney transplantation, hemodialysis, or peritoneal dialysis; ② abnormalities in immune system or coagulation function; ③ receipt of parenteral nutrition support; ④ concomitant other systemic diseases or malignant tumors; and ⑤ cognitive impairment or consciousness disorders.

Baseline eGFR was calculated or extracted from the admission medical records and was used to determine CKD stage. Primary renal disease was recorded according to the discharge diagnosis in the medical record. The primary reason for hospitalization was categorized according to the main admission reason recorded in the medical record, including CKD evaluation and treatment, edema/proteinuria management, blood pressure fluctuation, and medication adjustment. Length of hospital stay was defined as the number of days from admission to discharge. These variables were extracted because they may influence both nursing exposure and clinical outcomes.

Detailed renal biopsy-based histological diagnoses were not consistently available for all patients and were therefore not used as a stratification variable. Use of physician-prescribed immunosuppressive therapy during the index hospitalization or the 4-week follow-up period was also extracted and compared between groups. In this study, immunosuppressive therapy referred to medication prescribed for renal disease management, mainly in patients with nephrotic syndrome or chronic nephritis, and did not indicate a primary immune system abnormality. Patients with independent systemic autoimmune diseases, malignant tumors, or clinically significant immune-system abnormalities unrelated to the renal diagnosis were excluded according to the exclusion criteria.

According to the documented nursing intervention received during hospitalization and post-discharge follow-up, patients were retrospectively classified into a control group (*n* = 27) and an observation group (*n* = 30). Routine nursing was the standard nursing care pathway for non-dialysis CKD patients in the department. The BCW-informed early rehabilitation nursing model was gradually incorporated into routine extended nursing practice after nursing staff training and the establishment of WeChat-based follow-up support. Patients were classified into the observation group when their nursing records documented exposure to the complete intervention package, including admission assessment, WeChat-based education, individualized dietary and exercise guidance, medication reminders, peer-support activities, and post-discharge follow-up support. Patients whose records documented routine nursing only were classified into the control group. The nursing pathway was not selected by the research team according to CKD stage, eGFR, primary renal disease, immunosuppressive therapy, nutritional status, expected prognosis, or laboratory results.

To reduce potential selection bias, all eligible patients during the study period were screened according to the same inclusion and exclusion criteria. Patients with incomplete or unreliable clinical and nursing records were excluded. In addition to demographic characteristics, clinically relevant baseline variables that could influence both intervention exposure and outcomes were extracted and compared between groups, including primary renal disease category, CKD stage, baseline eGFR, reason for hospitalization, length of hospital stay, use of immunosuppressive therapy, baseline dietary compliance, and baseline quality of life. However, because this was a retrospective non-randomized study, residual selection bias, confounding by indication, and unmeasured confounding could not be completely avoided.

### 2.2 Ethical statement

This study involving human participants was reviewed and approved by the Ethics Committee of Wuxi People’s Hospital Affiliated to Nanjing Medical University (Approval No. WX159791). The nursing interventions analyzed in this study were delivered as part of routine clinical nursing practice and were not prospectively assigned by the research team for study purposes. Written informed consent for the use of clinical data and publication of anonymized results was obtained from all participants. All procedures were conducted in accordance with the Declaration of Helsinki.

### 2.3 Methods

#### 2.3.1 Control group

Patients in the control group received routine nursing intervention, including initial assessment upon admission, bedside education for patients and their families by the responsible nurse, introduction to the ward environment and attending physician; basic care during hospitalization (ward environment and patient safety care), specialized nursing (edema care, kidney puncture, urine sample collection, etc.), group lectures (medication, diet, physical exercise, etc.), regular ward rounds, and psychological care; discharge instructions including precautions, medication adherence, sensible diet, regular monitoring of blood pressure and urine routine, scheduled follow-ups, and outpatient reviews, etc.

#### 2.3.2 Observation group

Patients in the observation group received early rehabilitation nursing based on the BCW theory, with specific measures outlined as follows.

##### 2.3.2.1 Pre-intervention preparation

An intervention team was established, including 1 nephrology specialist physician, 1 specialized nurse, and 2 nursing staff proficient in CKD-related nursing care. No registered dietitian or renal nutrition specialist was formally included in the intervention team. Therefore, the dietary component of the intervention was delivered as standardized CKD dietary education rather than individualized medical nutrition therapy. The education emphasized general salt reduction, avoidance of excessive dietary fat, selection of appropriate high-quality protein sources, and weight-management principles. The nursing protocol did not prescribe stage-specific quantitative protein-intake targets or formally calculate individual energy requirements, and no uniform numerical targets for sodium, potassium, phosphorus, or fluid intake were specified by the nursing team. Where patient-specific dietary precautions or restrictions were documented in the treating physician’s medical orders, nurses reinforced these instructions during inpatient education and post-discharge follow-up, with clinical review by the nephrology specialist physician when needed. Accordingly, the dietary component should be interpreted as standardized CKD nursing education with physician-directed adjustments when clinically indicated, rather than as a stage-specific individualized medical nutrition therapy program. A WeChat network platform was set up for extended services primarily focused on health education and follow-up support for non-dialysis CKD patients.

##### 2.3.2.2 Intervention plan formulation

The BCW-informed early rehabilitation nursing intervention was organized according to the COM-B model to improve daily self-management behaviors among non-dialysis CKD patients. The target behaviors included dietary management, medication adherence, symptom monitoring, blood pressure monitoring, appropriate physical activity, and timely communication with healthcare professionals. Because this was a retrospective analysis of routine clinical nursing practice, the intervention was not developed through a formal prospective BCW development process. Instead, COM-B principles were used pragmatically to classify common self-management barriers and to match them with feasible nursing activities.

The relationship between COM-B components and intervention activities was clarified as follows. First, capability-related barriers included insufficient CKD knowledge, difficulty understanding dietary protein and salt control, limited ability to recognize symptoms or adverse drug reactions, and inadequate skills in blood pressure monitoring and urine specimen collection. These barriers were addressed mainly through education and training, including CKD educational manuals, bedside teaching, dietary education, medication precautions, symptom-recognition education, blood pressure monitoring instruction, urine specimen collection guidance, and individualized exercise guidance.

Second, opportunity-related barriers included limited access to continuous post-discharge guidance, insufficient communication channels with healthcare professionals, and lack of peer or family support. These barriers were addressed through environmental restructuring and enablement, including the WeChat-based follow-up platform, clarification of contact methods with healthcare professionals, medication and follow-up reminders, family participation, weekly follow-up contacts, and peer-support activities.

Third, motivation-related barriers included anxiety, low confidence in self-management, and fatalistic beliefs such as “CKD progression is inevitable.” These barriers were addressed through education, persuasion, modeling, and enablement, including admission-day motivation-building communication, goal setting, explanation that CKD progression may be delayed through appropriate management, peer experience-sharing, and encouragement of confidence in disease self-management.

The selected intervention functions were therefore education, training, persuasion, modeling, environmental restructuring, and enablement. Their selection was based on pragmatic APEASE considerations, including acceptability to patients and families, practicability within routine nursing work, affordability using existing staff and WeChat-based support, safety, and equity of access. Restriction was not included because no specific restrictive intervention was implemented in this study.

##### 2.3.2.3 Implementation of intervention

The BCW-informed early rehabilitation nursing intervention was delivered from admission to 4 weeks after discharge. During hospitalization, patients received three structured face-to-face education sessions. The first session was conducted on the day of admission and focused on assessment, explanation of disease-management goals, and motivation-building. The second and third sessions were conducted on days 2–3 of hospitalization and focused on CKD self-management education, including diet, medication precautions, symptom monitoring, blood pressure monitoring, urine specimen collection, and exercise guidance. Before discharge, individualized discharge guidance was provided.

After discharge, patients received WeChat-based follow-up support for 4 weeks. Health education messages related to CKD self-management were sent twice weekly. Medication reminders, follow-up reminders, and responses to patient questions were provided through the WeChat platform. In addition, one peer-support health education salon was conducted in the second week after discharge. Therefore, each patient in the observation group received at least four structured face-to-face or group education/support sessions, in addition to 4 weeks of WeChat-based follow-up support.

Participant engagement was monitored using routine nursing records. Nurses recorded whether patients or family members joined the WeChat group, attended the peer-support session, responded to weekly follow-up contacts, provided medication reminder feedback, reported dietary and blood pressure monitoring, and submitted questions or feedback through WeChat. The WeChat group was checked at least once weekly by the responsible nurse. Patients who did not respond to follow-up messages were contacted by telephone or during outpatient review when applicable. These records were used retrospectively to confirm exposure to BCW-informed nursing activities and to describe intervention engagement.

The contact intensity between groups was not matched. The control group received routine ward-based education, inpatient nursing care, discharge instructions, and scheduled outpatient review, whereas the observation group received additional structured education sessions, WeChat-based follow-up, medication and follow-up reminders, individualized guidance, and peer-support activities. Therefore, the comparison in this study should be interpreted as routine nursing versus a BCW-informed intensive nursing support package, rather than as an isolated test of the specific theoretical effect of BCW/COM-B components.

### 2.4 Observation indicators

Timing of outcome assessment: Baseline assessments were obtained at admission before the implementation of the documented nursing intervention. Post-intervention assessments were obtained after completion of the 4-week post-discharge follow-up period. Therefore, the “after intervention” measurements reflected the end of the complete nursing exposure period, including inpatient education, discharge guidance, WeChat-based follow-up, and the peer-support health education salon conducted in the second week after discharge. For the control group, post-intervention outcomes were collected at the corresponding 4-week post-discharge follow-up time point. Laboratory indicators were extracted from the outpatient review or follow-up laboratory records closest to the 4-week post-discharge assessment, and questionnaire outcomes were extracted from the follow-up nursing records at the same time point. Nutrition-related indicators and self-reported dietary adherence measured using the adapted RABQ were considered the main outcomes of interest, whereas renal function-related indicators, SF-36 total score, and reported nursing satisfaction were considered exploratory outcomes.

(1) Nutrition-related indicators: ALB, PA, and TF were obtained from routine morning fasting laboratory assessments at baseline and at the post-intervention follow-up assessment. BMI was calculated from height and body weight documented in the clinical records. Baseline body weight was obtained from the routine assessment at admission, and post-intervention body weight was obtained from the corresponding 4-week post-discharge follow-up record. Because this was a retrospective study, standardized information regarding edema severity, hydration status, body composition, clothing, time of day, and other conditions surrounding body-weight measurement was not consistently available. Accordingly, ALB, PA, TF, and BMI were analyzed as nutrition-related indicators rather than as validated direct measures of nutritional status.
(2) Levels of renal function-related indicators: before and after the intervention, 3 mL of morning fasting venous blood samples were collected from patients and analyzed using a fully automated biochemical analyzer to determine the levels of Blood Urea Nitrogen (BUN), Serum Creatinine (Scr), and Cystatin C (CysC).
(3) Self-reported dietary adherence assessment (8): before and after the intervention, self-reported dietary adherence was assessed using an adapted Chinese version of the Renal Adherence Behavior Questionnaire (RABQ) (8). The original RABQ was developed for hemodialysis patients as a 25-item self-report measure of renal dietary and fluid adherence. In the present study, the instrument was used as an exploratory measure in non-dialysis CKD patients rather than as a fully validated non-dialysis CKD-specific adherence instrument. Before use, the nephrology nursing team reviewed the Chinese wording and item content for linguistic clarity, relevance to local dietary habits, and applicability to non-dialysis CKD dietary and fluid self-management. All 25 original items were retained, and no items were deleted or newly added. The adaptation was limited to Chinese-language wording and contextual interpretation for non-dialysis CKD care; items containing hemodialysis-specific wording were retained only insofar as they reflected dietary or fluid self-management and were not interpreted as measures of adherence to dialysis treatment itself. The adapted questionnaire contained 25 items scored from 1 to 5, with higher scores indicating greater self-reported dietary adherence. Patients completed the questionnaire independently during routine nursing assessment or follow-up whenever possible. For patients with reading difficulty, trained nursing staff read the items verbatim and recorded the patient’s own response without interpretation or guidance. The questionnaire was administered as part of routine nursing follow-up, and nursing staff were not prospectively blinded to group allocation. In the present sample, the adapted RABQ showed acceptable internal consistency (Cronbach’s α = 0.87); however, internal consistency alone does not establish construct validity or criterion validity, and these forms of validity were not formally established for the adapted instrument in non-dialysis CKD patients. Accordingly, RABQ results were interpreted as exploratory self-reported adherence scores rather than as objective evidence of dietary compliance.
(4) Quality of life (9): before and after the intervention, the SF-36 Health Survey was used to assess health-related quality of life. The SF-36 comprises 8 dimensions, including physical functioning, role limitations due to physical health, bodily pain, general health perceptions, vitality, social functioning, role limitations due to emotional problems, and mental health. In this retrospective study, only the SF-36 total score recorded in the routine follow-up records was analyzed as an exploratory overall measure of health-related quality of life. Domain scores and physical or mental component summary scores were not available for all patients and were therefore not analyzed separately. Scores range from 0 to 100, with higher scores indicating better overall health-related quality of life.
(5) Nursing satisfaction: reported nursing satisfaction was assessed using the hospital’s self-made “Satisfaction Survey Questionnaire” during routine follow-up. The questionnaire consists of 20 questions, each rated from 1 to 5 according to satisfaction with nursing services. The total score ranges from 20 to 100, with scores below 70 indicating dissatisfaction, scores of 70 to 89 indicating satisfaction, and scores of 90 or above indicating very high satisfaction. This questionnaire had not undergone formal psychometric validation and was therefore used only as an exploratory measure of reported nursing satisfaction.

Outcome assessment and blinding: Because this was a retrospective, non-randomized observational study based on routine clinical nursing records, no prospective blinded outcome assessment was implemented at the time of care delivery. Objective laboratory outcomes, including ALB, PA, TF, BUN, Scr, and CysC, were extracted from the hospital laboratory information system. BMI was calculated from recorded height and weight. Dietary compliance, quality of life, and nursing satisfaction were derived from routinely collected questionnaire records. During retrospective data extraction, patient identifiers were removed, group information was coded, and the extracted dataset was checked by two researchers. Discrepancies were resolved by rechecking the original records. However, because patients and clinical nurses were aware of the nursing care received, blinding was not feasible for subjective outcomes, particularly nursing satisfaction.

### 2.5 Statistical analysis

GraphPad Prism 8 was used for graphical representation, and SPSS 22.0 was used for statistical analysis. Continuous variables were expressed as mean ± standard deviation, and categorical variables were expressed as frequency and percentage. Baseline between-group comparisons of continuous variables were performed using independent-samples *t* tests, and categorical variables were compared using chi-square tests or Fisher’s exact tests, as appropriate. Nutrition-related indicators and self-reported dietary adherence measured using the adapted RABQ were considered the main outcomes of interest, whereas renal function-related indicators, SF-36 total score, and reported nursing satisfaction were considered exploratory outcomes. Given the retrospective study design and the absence of a prospectively prespecified confirmatory endpoint, the statistical analyses were considered exploratory and hypothesis-generating rather than confirmatory. For outcomes measured before and after the intervention, within-group changes were calculated as post-intervention values minus baseline values. The primary comparative analysis focused on between-group differences in changes from baseline. Between-group comparisons of change values were performed using independent-samples t tests, and paired-samples t tests were used only to describe within-group changes. Post-intervention between-group comparisons were also reported as supportive descriptive analyses. Mean differences (MDs), 95% confidence intervals (CIs), and effect sizes were calculated where applicable to improve interpretability. Effect sizes for continuous outcomes were expressed as Hedges’ g. Exploratory baseline-adjusted sensitivity analyses were performed for the main continuous outcomes using analysis of covariance, with the post-intervention value as the dependent variable, group as the independent variable, and the corresponding baseline value as a covariate. Because of the limited sample size, adjustment for multiple clinical confounders was limited and the adjusted analyses were considered exploratory. For nursing satisfaction, because one cell contained zero patients, Fisher’s exact test was used for the comparison of total satisfaction after dichotomizing responses as dissatisfied versus satisfied/very satisfied. Because this was a retrospective exploratory study based on all eligible cases available during the study period, no *a priori* sample size or statistical power calculation was performed. Multiple clinically relevant outcomes were analyzed without formal adjustment for multiple comparisons, which may increase the risk of type I error. Therefore, the reported *p* values should be interpreted as nominal, unadjusted *p* values, and the findings should be considered exploratory and hypothesis-generating rather than confirmatory. A two-sided *p* value < 0.05 was considered statistically significant.

## 3 Results

### 3.1 Baseline data comparison

No statistically significant differences were observed between the two groups in the measured baseline demographic and clinical characteristics, including gender, age, primary disease, marital status, education level, CKD stage, baseline eGFR, reason for hospitalization, length of hospital stay, baseline RABQ score, and baseline SF-36 score (all *p* > 0.05). Specifically, the distribution of CKD stages was similar between the control group and the observation group, with 6 versus 7 patients in stage 1, 9 versus 10 patients in stage 2, 10 versus 11 patients in stage 3, 2 versus 2 patients in stage 4, and no stage 5 patients in either group. Baseline eGFR was also similar between the two groups (67.42 ± 18.73 vs. 66.85 ± 18.41 mL/min/1.73 m^2^, *p* = 0.908). Regarding hospitalization-related variables, the main reasons for hospitalization were CKD evaluation and treatment (12 vs. 13 patients), edema/proteinuria management (8 vs. 9 patients), blood pressure fluctuation (5 vs. 6 patients), and medication adjustment (2 vs. 2 patients), with no significant difference between groups (*p* = 0.998). The length of hospital stay was also comparable between the control and observation groups (8.41 ± 2.36 vs. 8.67 ± 2.49 days, *p* = 0.687). Use of immunosuppressive therapy was also comparable between groups, with 5 patients in the control group and 6 patients in the observation group receiving physician-prescribed immunosuppressive therapy for renal disease management during the index hospitalization or 4-week follow-up period (Fisher’s exact test, *p* = 1.000). Among these patients, the recorded indications were nephrotic syndrome or chronic nephritis. Please refer to Table 1 for details. However, because this was a retrospective non-randomized study, the absence of statistically significant differences in measured baseline variables should not be interpreted as evidence of baseline equivalence or as complete elimination of selection bias or unmeasured confounding.

**Table 1:** Baseline demographic and clinical characteristics of the two groups.

Variable	Control (*n* = 27)	Observation (*n* = 30)	Statistic	*p*
Gender			0.000	0.977
Male	17	19		
Female	10	11		
Age (years)	62.13 ± 8.65	62.72 ± 8.54	0.258	0.796
Primary disease			0.019	0.888
Chronic nephritis	13	15		
Nephrotic syndrome	7	8		
Hypertensive nephropathy	5	6		
Others	2	1		
Marital status			0.198	0.656
Yes	22	23		
No	5	7		
Education level			0.281	0.595
High school and below	21	25		
College and above	6	5		
CKD stage			0.477	0.924
Stage 1	6	7		
Stage 2	9	10		
Stage 3	10	11		
Stage 4	2	2		
Stage 5	0	0		
Baseline eGFR (mL/min/1.73 m^2^)	67.42 ± 18.73	66.85 ± 18.41	0.116	0.908
Reason for hospitalization			0.032	0.998
CKD evaluation and treatment	12	13		
Edema/proteinuria management	8	9		
Blood pressure fluctuation	5	6		
Medication adjustment	2	2		
Length of hospital stay (days)	8.41 ± 2.36	8.67 ± 2.49	0.405	0.687
Baseline RABQ score	68.43 ± 8.49	68.52 ± 8.23	0.040	0.967
Baseline SF-36 score	73.98 ± 6.54	74.27 ± 6.29	0.171	0.865
Use of immunosuppressive therapy			0.000	1.000
Yes	5	6		
No	22	24		

Values are presented as mean ± SD for continuous variables and n for categorical variables. CKD, chronic kidney disease; eGFR, estimated glomerular filtration rate; RABQ, Renal Adherence Behavior Questionnaire; SF-36, 36-item Short Form Health Survey. Continuous variables were compared using independent-samples *t* tests, and categorical variables were compared using chi-square tests or Fisher’s exact tests, as appropriate.

### 3.2 Comparison of nutrition-related indicators

As shown in Figure 1, the ALB levels for the control group before and after intervention were (30.59 ± 5.12, 31.17 ± 4.82), PA levels were (2.67 ± 0.13, 2.98 ± 0.19), TF levels were (3.42 ± 0.69, 3.47 ± 0.76), and BMI levels were (22.63 ± 2.07, 24.08 ± 2.16). For the observation group, before and after intervention, ALB levels were (31.03 ± 4.72, 37.23 ± 5.19), PA levels were (2.65 ± 0.15, 3.34 ± 0.23), TF levels were (3.39 ± 0.72, 3.72 ± 0.59), and BMI levels were (22.74 ± 2.05, 25.89 ± 3.11). Before the intervention, there were no significant between-group differences in ALB, PA, TF, or BMI (all *p* > 0.05). After the intervention, ALB, PA, and BMI were significantly higher in the observation group than in the control group (*p* < 0.001, *p* < 0.001, and *p* = 0.015, respectively), whereas TF was numerically higher but the between-group difference was not statistically significant (*p* = 0.169). Change-from-baseline analysis showed greater increases in nutrition-related indicators in the observation group than in the control group. The between-group difference in change was 5.62 g/L for ALB (95% CI: 2.80 to 8.44; Hedges’ g = 1.02), 0.38 g/L for PA (95% CI: 0.26 to 0.50; Hedges’ g = 1.60), 0.28 g/L for TF (95% CI: −0.10 to 0.66; Hedges’ g = 0.40), and 1.70 kg/m^2^ for BMI (95% CI: 0.25 to 3.15; Hedges’ g = 0.61). Exploratory baseline-adjusted ANCOVA showed adjusted between-group differences in post-intervention values of 5.87 g/L for ALB (95% CI: 3.43 to 8.30; *p* < 0.001), 0.37 g/L for PA (95% CI: 0.26 to 0.48; *p* < 0.001), 0.26 g/L for TF (95% CI: −0.06 to 0.58; *p* = 0.106), and 1.76 kg/m^2^ for BMI (95% CI: 0.41 to 3.11; *p* = 0.012), after adjustment for the corresponding baseline values. Because standardized edema, hydration-status, and body-composition data were not available at both assessments, the observed BMI change should be interpreted as a change in recorded BMI rather than as direct evidence of improved nutritional status.

**Figure 1:**
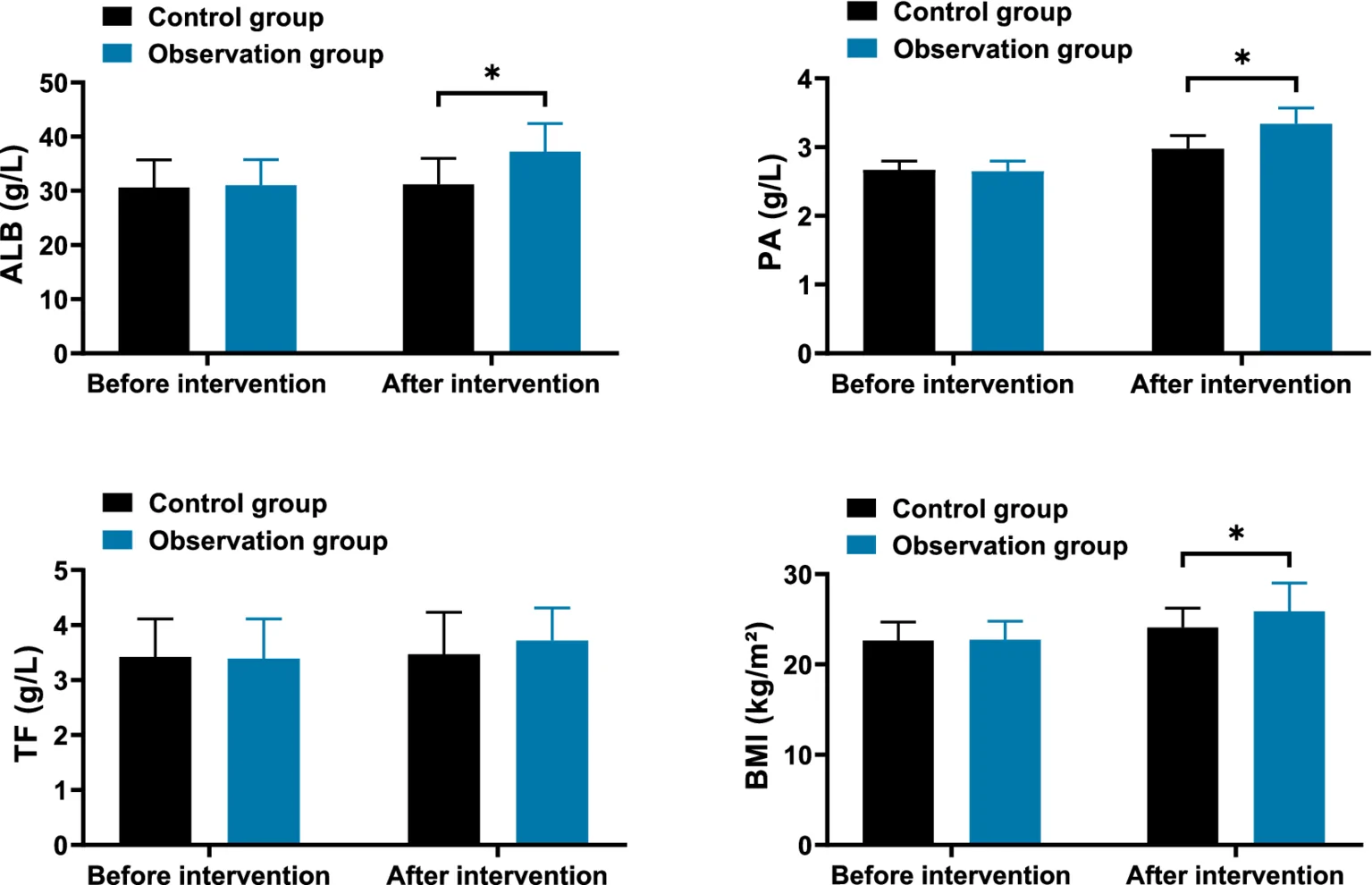
Comparison of nutrition-related indicators before and after intervention. Data are presented as mean ± SD. ALB, albumin; PA, prealbumin; TF, transferrin; BMI, body mass index. Error bars represent SD. *p < 0.05 for between-group comparison at the same time point.

### 3.3 Comparison of renal function-related index levels

As depicted in Figure 2, in the control group, BUN levels before and after the intervention were 5.53 ± 0.51 and 8.56 ± 1.13 mmol/L, Scr levels were 81.94 ± 20.07 and 88.43 ± 12.25 μmol/L, and CysC levels were 0.92 ± 0.19 and 0.84 ± 0.11 mg/L, respectively. In the observation group, BUN levels before and after the intervention were 5.56 ± 0.48 and 12.15 ± 1.07 mmol/L, Scr levels were 82.52 ± 19.14 and 94.26 ± 10.09 μmol/L, and CysC levels were 0.93 ± 0.17 and 0.76 ± 0.16 mg/L, respectively. Before the intervention, there were no significant differences in BUN, Scr, or CysC levels between the two groups (*p* > 0.05). After the intervention, BUN was significantly higher in the observation group than in the control group (*p* < 0.001), whereas CysC was significantly lower (*p* = 0.034). Scr was numerically higher in the observation group, but the between-group difference did not reach statistical significance (*p* = 0.054). These findings indicate that renal function-related indicators changed inconsistently and should not be interpreted as direct evidence of overall renal function improvement. In addition, because dietary protein intake, nPCR/nPNA, hydration status, and longitudinal eGFR were not available, the higher BUN level should not be used to infer improved dietary quality or improved renal function. Change-from-baseline analysis showed that the between-group difference in change was 3.56 mmol/L for BUN (95% CI: 2.94 to 4.18; Hedges’ g = 3.05), 5.25 μmol/L for Scr (95% CI: −1.45 to 11.95; Hedges’ g = 0.44), and −0.09 mg/L for CysC (95% CI: −0.17 to −0.01; Hedges’ g = −0.55). These change-score results were consistent with the post-intervention comparison and further support a cautious interpretation of renal function-related findings. Accordingly, all renal function-related analyses in this study should be regarded as exploratory.

**Figure 2:**
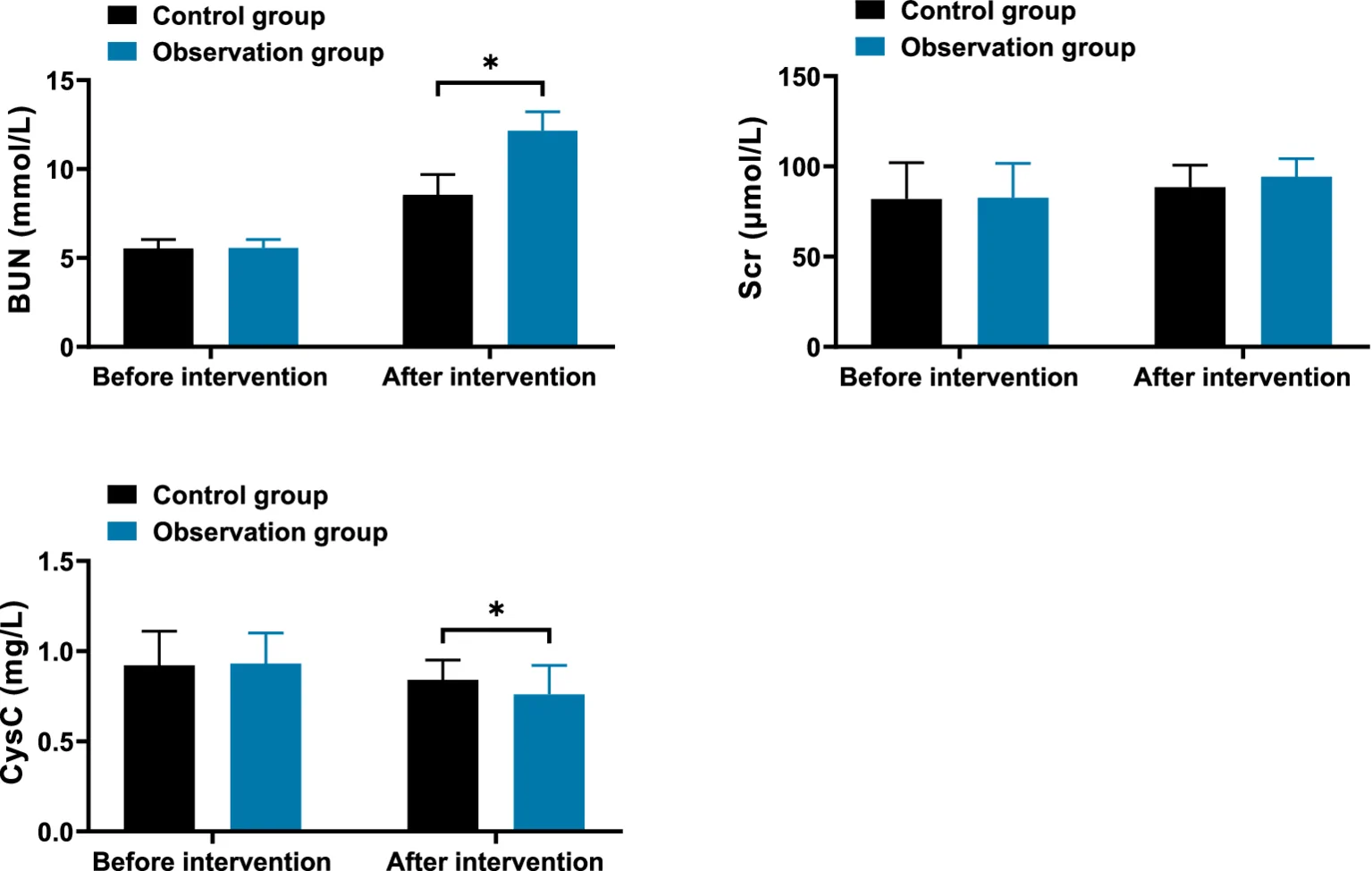
Comparison of renal function-related indicators before and after intervention. Data are presented as mean ± SD. BUN, blood urea nitrogen; Scr, serum creatinine; CysC, cystatin C. Error bars represent SD. *p < 0.05 for between-group comparison at the same time point.

### 3.4 Comparison of self-reported dietary adherence

Before the intervention, there was no significant difference in adapted RABQ scores between the two groups (*p* > 0.05); however, after the intervention, the observation group had significantly higher adapted RABQ scores than the control group (*p* < 0.05), indicating higher self-reported dietary adherence as measured by this adapted instrument. The mean increase in RABQ score from baseline was 14.82 points in the control group and 28.64 points in the observation group. The between-group difference in change was 13.82 points (95% CI: 8.19 to 19.45; Hedges’ g = 1.28). Exploratory baseline-adjusted ANCOVA showed an adjusted between-group difference of 13.87 points in the post-intervention adapted RABQ score (95% CI: 8.76 to 18.98; *p* < 0.001) after adjustment for baseline RABQ score. These findings reflect changes in self-reported adherence scores and should not be interpreted as objectively verified changes in dietary intake or dietary compliance (Table 2).

**Table 2:** Comparison of self-reported dietary adherence assessed using the adapted RABQ.

Group (*n*)	RABQ scores	
	Before intervention	After intervention
Control (*n* = 27)	68.43 ± 8.49	83.25 ± 10.24
Observation (*n* = 30)	68.52 ± 8.23	97.16 ± 10.19
*t*	0.040	5.133
*p*	0.967	<0.001

Values are presented as mean ± SD. RABQ, Renal Adherence Behavior Questionnaire. The adapted RABQ was used as an exploratory self-report measure of dietary adherence and was not formally validated for non-dialysis CKD patients. Higher scores indicate greater self-reported adherence. Between-group comparisons at each time point were performed using independent-samples t tests. Change-from-baseline differences, 95% CIs, and Hedges’ g are reported in the text.

### 3.5 Comparison of SF-36 total scores

Before the intervention, there was no significant difference in SF-36 total scores between the two groups (*p* > 0.05). After the intervention, the observation group had a higher SF-36 total score than the control group (*p* < 0.05), as detailed in Table 3. The mean increase in SF-36 total score from baseline was 4.98 points in the control group and 9.58 points in the observation group. The between-group difference in change was 4.60 points (95% CI: 0.94 to 8.26; Hedges’ g = 0.62). Exploratory baseline-adjusted ANCOVA showed an adjusted between-group difference of 4.76 points in the post-intervention SF-36 total score (95% CI: 1.63 to 7.90; *p* = 0.004) after adjustment for baseline SF-36 total score. Because SF-36 domain scores and component summary scores were not analyzed, this finding should be interpreted as an exploratory overall quality-of-life result rather than evidence of domain-specific improvement.

**Table 3:** Comparison of SF-36 total scores.

Group (*n*)	SF-36 total score	
	Before intervention	After intervention
Control (*n* = 27)	73.98 ± 6.54	78.96 ± 6.43
Observation (*n* = 30)	74.27 ± 6.29	83.85 ± 6.51
*t*	0.171	2.848
*p*	0.865	0.006

Values are presented as mean ± SD. SF-36, 36-item Short Form Health Survey. Only the SF-36 total score was analyzed in this study. Between-group comparisons at each time point were performed using independent-samples t tests. Change-from-baseline differences, 95% CIs, and Hedges’ g are reported in the text.

### 3.6 Comparison of nursing satisfaction

The reported nursing satisfaction rate was 77.78% in the control group and 100.00% in the observation group. Because one cell contained zero patients, total satisfaction was compared using Fisher’s exact test after dichotomizing responses as dissatisfied versus satisfied/very satisfied. The total satisfaction rate was 22.22 percentage points higher in the observation group than in the control group, with a risk difference of 22.22% (95% CI: 5.98 to 40.76%; Fisher’s exact test, *p* = 0.008), as detailed in Table 4. Because the satisfaction questionnaire was locally developed and had not undergone formal psychometric validation, outcome assessment was not blinded, and the observation group received greater nursing contact and support, the 100.00% reported satisfaction rate should be regarded as an exploratory patient-reported outcome and should not be interpreted as strong evidence of intervention effectiveness.

**Table 4:** Comparison of nursing satisfaction.

Group	*n*	Dissatisfied	Satisfied	Very satisfied	Total satisfaction rate (%)
Control	27	6	15	6	77.78%
Observation	30	0	17	13	100.00%
Fisher’s exact test	-	-	-	-	-
*p*	-	-	-	-	0.008

Values are presented as *n*. Total satisfaction was dichotomized as dissatisfied versus satisfied/very satisfied for statistical comparison. Because one cell contained zero patients, Fisher’s exact test was used. The risk difference and 95% CI are reported in the text.

## 4 Discussion

The BCW provides a useful theoretical framework for organizing self-management-oriented health education and nursing support. Patients manage their health through self-effort, leading to disease control and promoting personal health (10). Previous studies (11, 12) have emphasized patient-centered, self-management-based health education as an important component of rehabilitation nursing. Hence, this study tailored a health education rehabilitation nursing model suitable for non-dialysis CKD patients by reconstructing and shaping patient motivation, ability, and opportunities. By focusing on patients’ subjective initiative, it strengthens their belief in disease control and coping strategies, establishing a patient-centered, dynamic, and interactive health education nursing intervention model.

Previous research (13) has shown that effective self-management is associated with better nutritional status in non-dialysis CKD patients. Nutritional status is an important factor affecting the prognosis of CKD patients, and appropriate nutritional management may help support quality of life and clinical outcomes (14). ALB, PA, TF, and BMI are commonly reported as nutrition-related indicators; however, these measures are not specific markers of nutritional status and may also be influenced by clinical factors other than dietary intake. In this study, non-dialysis CKD patients in the observation group had more favorable post-intervention nutrition-related indicators than those in the control group. This finding is broadly consistent with the CKD nutrition literature indicating that dietary management and protein-energy wasting are closely related to nutritional status and clinical outcomes (15, 16). CKD-related metabolic disturbances, including abnormalities in water, electrolyte, and acid–base balance, may also affect appetite, nutrient intake, and body-fluid status (17). Nevertheless, the magnitude of the short-term changes observed in the present study, particularly the increase in BMI, requires cautious interpretation. In patients with CKD, short-term changes in body weight and BMI may reflect changes in extracellular fluid volume or edema as well as changes in body mass. Similarly, ALB, PA, and TF may be influenced by hydration status, inflammation, protein losses, liver function, and underlying disease activity in addition to nutritional intake. Because standardized edema and hydration assessments, body-composition measurements, and objective dietary-intake data were not available at both assessment points, the observed increases in BMI and ALB cannot be attributed specifically to improvement in nutritional status. Accordingly, these findings should be interpreted as changes in nutrition-related indicators rather than evidence of a confirmed improvement in nutritional status.

However, the renal function-related markers did not change in a consistent direction: BUN and Scr were higher in the observation group after the intervention, whereas CysC was lower. Although increased BUN may be related to increased protein intake in some clinical contexts, this explanation cannot be confirmed in the present study because dietary protein intake, nPCR/nPNA, hydration status, longitudinal eGFR, and longitudinal information on muscle mass were not available. In addition, BUN and Scr may be influenced by renal excretory function, hydration status, catabolic state, muscle mass, medication changes, and underlying disease activity. Because these factors were not comprehensively characterized at both assessment points, the mechanisms underlying the discordant changes in BUN, Scr, and CysC cannot be determined, and no renal benefit can be inferred from these findings. Therefore, BUN should not be interpreted as evidence of improved dietary quality, and the renal function-related findings should be regarded as exploratory clinical indicators rather than evidence that the intervention improved renal function. Future studies should include dietary intake assessment, nPCR/nPNA where applicable, repeated eGFR measurements, hydration assessment, and longer follow-up to clarify renal outcomes.

The BCW-informed intensive nursing support package was associated with higher self-reported dietary adherence scores measured using the adapted RABQ, higher quality-of-life scores, and higher reported nursing satisfaction among non-dialysis CKD patients. However, the observation group also received more frequent and structured nursing contact, including WeChat-based follow-up, reminders, peer support, and individualized guidance. Therefore, the observed differences in subjective outcomes may reflect the combined influence of BCW-informed content, increased contact intensity, and greater nursing attention, rather than the specific effect of BCW/COM-B mechanisms alone. As the condition progresses, the physiological burden on CKD patients gradually increases, often leading to various complications in the later stages (18). CKD exhibits typical characteristics of chronic non-communicable diseases, lingering with difficulty in cure, closely linked to lifestyle habits, significantly impacting the quality of life (19). In this study, non-dialysis CKD patients in the observation group had higher RABQ scores, quality-of-life scores, and reported nursing satisfaction than those in the control group. This suggests that the BCW-informed intensive nursing support package may be associated with higher self-reported dietary adherence scores on the adapted RABQ and better quality-of-life scores, as well as higher reported nursing satisfaction among non-dialysis CKD patients. This finding is broadly consistent with the self-management experiences reported by Baay et al. (20). More broadly, previous nursing studies in adult rehabilitation and chronic disease populations have also reported the potential value of structured rehabilitation nursing or integrated nursing care in supporting functional recovery and care-related outcomes (21, 22). The BCW-informed intensive nursing support package included management and support related to diet, disease, appropriate exercise, follow-up, and psychological aspects. It was designed to provide WeChat-based follow-up support, encourage patient participation, and support confidence in disease self-management, which may be related to better patient-reported outcomes. On the other hand, in the mid-to-late stages of CKD, patients often experience multiple complications such as electrolyte imbalances, renal anemia, and calcium-phosphorus metabolism disorders, leading to symptoms like nausea, vomiting, dizziness, fatigue, and bone pain, directly impacting the patient’s dietary compliance and quality of life. The dietary component of the BCW-informed intensive nursing support package included general salt-reduction and low-fat dietary education, qualitative guidance on appropriate high-quality protein choices, and practical meal-planning and weight-management guidance, alongside medication reminders, blood pressure monitoring guidance, condition monitoring, urine sample collection education, and individualized exercise planning. However, it did not constitute stage-specific medical nutrition therapy and did not include formal quantitative prescriptions for protein or energy intake or uniform numerical targets for sodium, potassium, phosphorus, or fluid intake.

Although this study yielded some positive findings regarding the association between BCW-informed early rehabilitation nursing and compliance-related outcomes among non-dialysis CKD patients, several limitations should be acknowledged. First, this was a retrospective, non-randomized observational study with a relatively small sample size of 57 patients, including 27 patients in the control group and 30 patients in the observation group. Patients were classified according to the nursing interventions documented in their clinical and nursing records rather than being randomly assigned by the research team. Although we supplemented the baseline comparison by adding primary renal disease category, CKD stage, baseline eGFR, reason for hospitalization, length of hospital stay, use of immunosuppressive therapy, baseline dietary compliance, and baseline quality of life, these measured variables cannot fully establish comparability between groups. Detailed renal biopsy-based histological diagnoses, specific immunosuppressive medication regimens, doses, and treatment duration were not uniformly available; therefore, residual confounding related to underlying renal pathology or treatment intensity could not be excluded. In addition, because the number of patients with advanced CKD was small and no stage 5 patients were included, stage-specific subgroup analysis or robust adjustment by CKD severity could not be performed. Residual selection bias, confounding by indication, and unmeasured confounding related to disease severity, clinical stability, nutritional risk, inflammatory status, comorbidity burden, medication changes, socioeconomic status, actual dietary intake, motivation, access to care, or nursing exposure could not be completely excluded. Accordingly, the present findings should be interpreted as associations rather than causal effects. In addition, standardized assessments of edema and hydration status and body-composition measurements were not available at both assessment points. Therefore, short-term changes in recorded body weight and BMI could not be distinguished from changes related to extracellular fluid status, and changes in ALB and other nutrition-related biomarkers cannot be attributed solely to nutritional improvement. Second, renal function-related assessment was limited. Renal function was evaluated only using BUN, Scr, and CysC, while longitudinal eGFR, dietary protein intake, nPCR/nPNA, standardized hydration assessment, longitudinal muscle-mass information, detailed medication changes, catabolic status, and disease-activity measures were not consistently available in this retrospective dataset. Therefore, the mechanisms underlying the discordant changes in BUN, Scr, and CysC could not be determined, because these biomarkers may be influenced by renal excretory function, nutritional intake, hydration status, muscle mass, catabolism, medications, and underlying disease activity. The renal function-related findings should therefore be regarded as exploratory and should not be used to infer improved dietary quality, renal function improvement, or a renal benefit of the intervention. Third, the statistical analysis was limited by the small sample size and exploratory nature of the study. No *a priori* sample size or power calculation was performed, and multiple outcomes were analyzed without formal adjustment for multiple comparisons. Although change-from-baseline differences, MDs, 95% CIs, effect sizes, and exploratory baseline-adjusted sensitivity analyses were added to improve interpretability, the retrospective design and limited sample size restricted the ability to perform robust multivariable adjustment for clinical confounders. Therefore, these findings should be considered exploratory and hypothesis-generating rather than confirmatory, particularly given the absence of formal adjustment for multiple comparisons. Fourth, dietary compliance and dietary intervention-related findings should be interpreted with caution. The RABQ was originally developed and validated in hemodialysis patients, and no fully validated Chinese non-dialysis CKD-specific version was used in this study. Although the adapted Chinese version showed acceptable internal consistency in the present sample (Cronbach’s α = 0.87), internal consistency alone does not establish construct validity or criterion validity. The adapted instrument did not undergo formal cross-cultural adaptation or comprehensive psychometric validation in non-dialysis CKD patients. Therefore, the RABQ findings should be interpreted as exploratory changes in self-reported dietary adherence measured using an adapted instrument rather than as objectively demonstrated dietary compliance, and measurement bias cannot be excluded. In addition, the dietary component was delivered by the nephrology physician and nursing team using standardized CKD nursing education materials, without formal involvement of a registered dietitian or renal nutrition specialist. Furthermore, participants spanned CKD stages 1–4, whereas the nursing protocol did not use prospectively standardized stage-specific quantitative prescriptions for protein or energy intake or uniform numerical targets for sodium, potassium, phosphorus, or fluid intake. Patient-specific dietary precautions were reinforced when documented in physician orders; therefore, the standardized nursing education may not have fully captured the heterogeneous nutritional requirements of patients at different CKD stages. Objective dietary intake data, such as protein intake, sodium intake, total energy intake, dietary pattern, urinary sodium excretion, or nPCR/nPNA, were not available; therefore, changes in dietary behavior and dietary quality could not be objectively verified. Fifth, subjective outcomes were vulnerable to bias. No prospective blinded outcome assessment was conducted. Although objective laboratory indicators were extracted from routine laboratory records and retrospective data extraction was performed using de-identified and coded data, subjective outcomes such as dietary compliance, SF-36 total score, and reported nursing satisfaction may still be affected by expectation bias, social desirability bias, response bias, or assessment bias. In addition, nursing satisfaction was assessed using a hospital-made questionnaire that had not undergone formal psychometric validation; therefore, the 100.00% reported satisfaction rate in the observation group should be interpreted cautiously. The SF-36 was analyzed using only the total score, while domain scores and physical or mental component summary scores were not available for all patients and were not analyzed separately, which limits the interpretability of quality-of-life findings. In addition, the study did not include an attention-matched control group. The observation group received more frequent and structured nursing contact than the control group, including WeChat-based follow-up, reminders, peer support, and individualized guidance. Therefore, differences in self-reported adherence, SF-36 total score, and reported nursing satisfaction cannot be clearly separated from nonspecific attention or intervention-dose effects, and the greater nursing contact received by the observation group represents a potential source of performance bias. Sixth, although the intervention was guided by the BCW and COM-B model, it did not involve a formal prospective COM-B behavioral diagnosis, structured APEASE scoring, or independent expert consensus process. Therefore, the theoretical mapping between BCW components and nursing activities should be regarded as pragmatic and preliminary. In addition, this study lacked long-term follow-up, so the sustainability of the observed associations between BCW-informed early rehabilitation nursing and patient outcomes remains unclear. The heterogeneity of non-dialysis CKD patients was not fully addressed, and subgroup differences related to disease stage, clinical characteristics, renal diagnosis, treatment regimen, or specific patient needs may have influenced the results. Finally, because this was a single-center study conducted in a specific clinical setting, the generalizability of the findings to other institutions, regions, or patient populations may be limited. Future prospective, multicenter studies with larger sample sizes, longer follow-up periods, formal BCW development procedures, attention-matched control groups, more comprehensive renal and dietary assessments, and adequate adjustment for confounding are needed to further verify these findings.

## 5 Conclusion

The BCW-informed intensive nursing support package was associated with more favorable nutrition-related indicators, higher self-reported dietary adherence scores measured using the adapted RABQ, better quality-of-life scores, and higher reported nursing satisfaction among non-dialysis CKD patients. However, because the study was retrospective and non-randomized and the observation group received greater nursing contact than the control group, these findings should be interpreted as associations rather than causal effects, and the specific contribution of the BCW/COM-B framework cannot be separated from nonspecific attention or intervention-dose effects. The renal function-related findings were inconsistent, as BUN and Scr were higher in the observation group after the intervention, whereas CysC was lower. Because dietary protein intake, nPCR/nPNA, hydration status, and longitudinal eGFR were not available, the data do not support a conclusion that the intervention improved renal function or dietary quality based on BUN changes. Further prospective studies with larger sample sizes, attention-matched control groups, dietary intake assessment, eGFR monitoring, hydration assessment, and adequate adjustment for confounding are needed to clarify the specific effects of BCW-informed nursing components and their clinical significance.

## Data Availability

The original contributions presented in the study are included in the article/supplementary material, further inquiries can be directed to the corresponding author/s.
